# Funding social innovation for health with research funds for development

**DOI:** 10.1186/s40249-020-00744-w

**Published:** 2020-08-27

**Authors:** Hannah Akuffo, Teresa Soop

**Affiliations:** Unit for Research Cooperation, Department for Partnerships, Swedish International Development Cooperation Agency, Sida. Valhallavägen 199, 105 25 Stockholm, Sweden

**Keywords:** Research capacity strengthening, Use of research, Social innovation, Research funding

## Abstract

Why and when is it appropriate and relevant to use research funds for social innovation to support both conventional scholarly researchers and non-researchers working in collaboration?

## Main text

The scarcity of research funding available in low-income countries, and the enormous needs of those countries, suggest that research for development money might be better spent to support qualified researchers who are, at least in theory, more likely to develop new research agendas, make new discoveries, and through the transfer of their research, stimulate innovation.

However, a cursory look at the many places of commerce in various African countries, including in crowded markets, provides a glimpse of the ingenuity and innovation that prevails in such settings.

Over many decades, Sida has provided support to strengthen research capacity in low income countries, with emphasis on “the use of the research”– defined here both as utilising local research expertise and utilising the important relevant research results obtained. This philosophy has fed into the initial steps of innovation and especially into achieving societal impact from this [1]. This need not occur in orthodox research settings. However, connecting research and innovation is a means to ensure that research results are used and enhance sustainable impact, with the goal to reduce poverty and build sustainable societies.

A systems approach to innovation that focuses on the creation of an enabling environment for innovation is thus promoted. A key feature for any successful innovation, especially in low income countries, is efficient and effective interaction between the different stakeholders at an early stage. This includes universities, the private sector, the public sector and civil society.

Sida has interpreted its role in part, to fund and to facilitate dialogue to harness important ideas and ways of approaching health care and the prevention of disease and ill health through local innovations. This is especially relevant when the solutions that derive from this approach are accessible by and affordable for people who are poor, or whose financial status is unpredictable. Social innovation procedures generated in local settings, in response to local social structures, contingencies and constraints, are more likely to be able to address these roadblocks in appropriate health delivery. Furthermore, finding methods that allow rigorous documentation of the innovation and assess its reproducibility, through the interactions of researchers with social innovators, has the potential to make such social innovations in health very powerful tools to improve health in low income settings.

Sida is committed to strengthening local institutional research capacity in low income countries and supporting organisations with a focus on social innovation, such as TDR where, in addition to its undesignated funding, Sida provides specific funding to support social innovation [2]. This opens the opportunity for social innovators to analyse and gather the evidence needed to ascertain the reproducibility of affordable social innovations in health. Such assessment is vital when making the case to policy and other decision makers who seek health innovations that can have positive impact on the health of a wide range of people, regardless of their financial status.

## Conclusions

The establishment of research hubs within the framework of the Social Innovation for Health Initiative, through competitive processes, provides the opportunity for prospective social innovators in various countries. The aim is to develop further skills to help simple, accessible and effective social innovations in health to be identified. By nurturing academic, community and institutional collaboration and communication, we are most likely to identify and target for implementation, interventions in the places where they can make the greatest difference.

## Data Availability

Not applicable.

## References

[1] Phills JA, Deiglmeir K, Miller DT. Rediscovering social innovation. Stand Soc Innov Rev. 2008;6(4):34–43.

[2] Halpaap B, Peeling RW, Bonnici F. The Role of Multilateral Organizations and Governments in Advancing Social Innovation in Health Care Delivery. Infect Dis Poverty. 2019;8(1):81. 10.1186/s40249-019-0592-y PMC6743093 31514738

